# Are Attitudes towards COVID-19 Pandemic Related to Subjective Physical and Mental Health?

**DOI:** 10.3390/ijerph192114538

**Published:** 2022-11-05

**Authors:** Kristine Khachatryan, Manfred E. Beutel, Yve Stöbel-Richter, Markus Zenger, Hendrik Berth, Elmar Brähler, Peter Schmidt

**Affiliations:** 1 Department of Psychosomatic Medicine and Psychotherapy, University Medical Center, Johannes Gutenberg-University Mainz, Untere Zahlbacher Str. 8, 55131 Mainz, Germany; 2 Department of Psychiatry and Psychotherapy, Leipzig University Medical Center, Semmelweisstr. 10, 04103 Leipzig, Germany; 3 Faculty of Managerial and Cultural Studies, University of Applied Sciences Zittau/Görlitz, Furstr. 3, 02826 Görlitz, Germany; 4 Department of Applied Human Studies, University of Applied Sciences Magdeburg-Stendal, Osterburger Str. 25, 39576 Stendal, Germany; 5 Integrated Research and Treatment Center Adiposity Diseases—Behavioral Medicine, Psychosomatic Medicine and Psychotherapy, University of Leipzig Medical Center, Philipp-Rosenthal-Strasse 27, 04103 Leipzig, Germany; 6 Division of Psychological and Social Medicine and Developmental Neurosciences, Research Group Medical Psychology and Medical Sociology, Medical Faculty Carl Gustav Carus, Technische Universität Dresden, Fetscherstr. 74, 01307 Dresden, Germany; 7 Department of Political Science and the Centre of International Development and Environment (ZEU), University of Giessen, Karl-Glöckner-Straße 21E, 35394 Giessen, Germany

**Keywords:** COVID-19 pandemic, COVID-19 anxiety, pandemic denial, longitudinal data, Germany

## Abstract

In this paper, we investigate the relationship between a person’s psychological distress, subjective physical health and their attitudes towards the COVID-19 pandemic. The evaluation was performed on the basis of data from two waves of the Saxon Longitudinal Study, carried out in 2019 (pre-pandemic) and 2021. The number of study participants in both waves was 291. We tested in autoregressive cross-lagged models the stability of the respondents’ health status before and during the pandemic and reviewed their influence on attitudes towards COVID-19. Our results show that COVID-19-related concerns are controlled by subjective physical health, while pandemic denial is linked to psychological distress. In an unknown and critical situation, with limited control over the situation, the strategy of avoidance or suppression may be used by individuals for protection by psychologically downplaying the stressor and danger.

## 1. Introduction

The Coronavirus Disease 2019 (COVID-19) pandemic has turned the world upside down. Worldwide, from January 2020 to July 2022 more than half a billion people have already been infected by COVID-19 and the number of deaths has already reached more than six million [1]. Nevertheless, there are more and more people who downplay the disease and its consequences or deny the pandemic.

Attitudes towards the pandemic can depend on several factors. The role of science and research in political decisions has probably never been as strongly addressed in the media as it has been during the pandemic. All decisions, especially on nonpharmaceutical interventions such as lockdowns and restrictions in public life, were attempted to be based on empirical evidence, although, for example, the German Representatives for Evidence Based Medicine (Sachverständigenausschuss) and the COVID EXPERT Panel summarized a lot of weaknesses and faults, especially missing data and evaluations [2]. However, scientific findings are unlikely to find a similar resonance in all sections of the population. For instance, people who believe in conspiracy theories might be ill-convinced by the scientific findings and have a hard time adapting their behavior accordingly. Consequences for individuals caused by restrictions in public and private life can also be decisive.

According to Lazarus and Cohen [3], major changes that are often cataclysmic and affect a large number of people are one of the types of environmental events called stress stimuli. While extreme environmental conditions can cause stress in almost anyone, there is a wide variation in individual responses to such universal stressors. The relationship between the environment and the person is meditated by two processes. The cognitive appraisal determines why and to what extent some interactions between the person and the environment are stressful. Through coping, one manages the demands of the environmental–person relationship that are appraised as stressful. The types of coping are distinguished depending on whether the coping is aimed at managing the problem or regulating the emotional response to the problem. With the appraisal that nothing can be changed in the situation, emotion-focused forms of coping usually occur. If the environmental conditions are considered as changeable, then problem-focused forms of coping are used more frequently [4,5]. Lazarus and Folkman [4] consider health and energy as a physical resource of coping that is particularly important in persistent stressful situations.

There is empirical evidence that COVID-19 affects not only physical but also mental health. Even after a milder course of the disease, there may be long-term serious consequences for health (see, e.g., [6,7]). Even the overall situation with the pandemic can affect the mental health of people. Several studies have found different effects of the pandemic on mental health, including depression, anxiety disorders, stress, emotional disturbance, etc. (see literature review carried out by Hossain et al. [5]). In this context, it would be expected that particularly people with impaired health should be afraid of becoming infected and realize the dangers of the pandemic. At the same time, the COVID-19 pandemic is a situation with a lot of uncertainty, especially at the beginning, so problem-focused forms of coping are not always applicable. In such circumstances, emotion-focused strategies could gain more importance, so that minimization, distancing, and selective attention could be used as appropriate responses to stress.

In this paper, we examine the research question of whether subjective physical and mental health influences attitudes towards the COVID-19 pandemic. We focus on perceived physical health and mental distress. For this purpose, we calculated autoregressive cross-lagged models with the data of the Saxon Longitudinal Study (SLS) [8]. The added value of our study is that through the longitudinal design of the SLS we can check the respondents’ health status of the pre-pandemic and during the pandemic in 2021 and then test its effects on current attitudes towards COVID-19. The data from the pre-pandemic period enabled us to check whether the current health status existed before the pandemic or may itself be a result of the pandemic. Furthermore, we tested the equivalence of the measurements over time.

### 1.1. Impact of the COVID-19 Pandemic on Human Health

The COVID-19 pandemic is having an impact not only on physical health, but also on the psyche. Symptoms such as fatigue, dyspnea, and loss of taste or smell are often reported after acute COVID-19 disease [9]. In addition, joint pain and chest pain occur more frequently [10]. Even after a mild course of the disease or unnoticed infection, longer-term health consequences can occur, which is called Long COVID or post-COVID-19 syndrome [11].

The systematic review and meta-analysis carried out by Salari et al. [12] shows that the prevalence rates of stress, anxiety, and depression, as a result of the pandemic in the general population of Asia and Europe, are 29.6%, 31.9% and 33.7%, respectively. The results of the meta-analysis show that people who followed COVID-19 news were more likely to be affected by anxiety. In particular, inconsistent information and misinformation can cause depressive symptoms in the population. In populations with less established health systems, the extent of psychological effects can become even greater. In terms of socio-demographic characteristics, it can be seen that women are more often affected by stress, anxiety, and depression than men. Although the mortality risk of COVID-19 is higher among elderly people, the group of 21–40-year-olds shows the highest levels of anxiety, depression and stress. According to Salari et al. [12], an explanation for this could be that this age group, comprising active workers in society, is more likely to be affected by the economic consequences of the pandemic and is worried about the future. Another explanation would be that young people have greater access to information through social media, which could also be a stressor. With regard to the role of work as a protective factor, a study from Pakistan shows that psychological distress is related to more significant death anxiety among non-working compared to those who are working [13]. High education is also associated with high levels of anxiety, stress and depression. In addition, health anxiety, own medical history, chronic diseases as well as having a COVID-19 patient in the family or circle of friends lead to increased anxiety and depression [12]. An online study on psychological distress in the pandemic from Ethiopia showed that respondents with high perceived severity had increased mental distress, and those with the highest score of perceived response efficacy had low distress [14].

The COVID-19 pandemic is often described as a collective traumatic stressor that can produce post-traumatic symptoms [15,16,17]. An online study conducted in Italy examined the consequences of the COVID-19 pandemic in the context of post-traumatic stress disorder (PTSD). The relationship of the three main symptoms of PTSD, intrusion, hyperarousal, and avoidance with fear of COVID-19 and mental health was investigated. The results show that intrusion correlates positively with fear of COVID-19 and negatively with mental health. Hyperarousal mediates the relationship between intrusion and fear of COVID-19 on the one hand and between intrusion and mental health on the other hand [16].

The systematic review and meta-analysis carried out by Kunzler et al. [18] shows that in the general population, an increase in anxiety and depression compared to pre-pandemic times can be observed, with anxiety having a small increase and depression having a moderate increase. Interestingly, similar findings have not been found in patients (e.g., COVID-19 patients, patients with pre-existing physical and mental conditions) or among medical personnel. However, an important factor is the choice of the period as a reference for the prevalence rates. The results of the meta-analysis of Kunzler et al. [18] show that anxiety and depressive symptoms in the population are much higher when compared with the data from five or more years before the pandemic than when compared with those from the pre-pandemic period of less than two years.

A study from China has examined the effects of quarantine in the pandemic on mental health. The results show that home self-quarantine is associated with a decrease in depression and an increase in happiness, while community-level quarantine is associated with decreased happiness [19].

With regard to Germany, the study by Entringer and Kröger [20] showed that people’s loneliness was still greatly increased in the second lockdown (January/February 2021) compared to the pre-pandemic level, but did not increase further compared to the first lockdown (March to July 2020). Depression and anxiety symptoms decreased slightly again in the second lockdown compared to the first lockdown and were comparable to the level in 2016 (similar results in [21]). It turns out that in particular women, younger people and people with a direct migration background suffered from the second lockdown: they reported higher loneliness, higher depression and anxiety symptoms, lower affective well-being and lower life satisfaction. At the same time, socio-economic factors such as education and income played a smaller role in differences in mental health and well-being than before the pandemic. The study by Entringer et al. [22] came to an interesting finding that in 2020 the satisfaction with health in Germany increased significantly across all population groups, while concerns about health fell significantly across all groups. According to the authors, this suggests that the current assessment was made strongly in the context of the threat scenario of the pandemic. There is also empirical evidence that a positive appraisal style was positively associated with mental resilience during the first lockdown in Europe [23].

While older people are particularly vulnerable to the pandemic, evidence can be found about the resilience of this group. A qualitative study from Germany showed that older people showed a predominantly stable condition and good coping with the COVID-19 events. Life experience, previously overcome crises, an optimistic attitude and insight into the necessity of the measures were mentioned as essential resources [24].

The evaluation of Liebig et al. [25] within the SOEP-CoV-Study showed that the post-reunification generation in East Germany (under 35-year-olds) was less lonely in 2020 and tended to rate stressful situations more positively than their peers in the West.

Empirical evidence has been found about social inequality in the pandemic. A study by Hoebel et al. [26] shows that from the second wave of the pandemic onwards, COVID-19 infections and deaths have increasingly shifted to socio-economically disadvantaged regions. People with low education have twice the risk of infection as people with high education. People from socio-economically disadvantaged groups and deprived regions are tested less and thus infections often go undetected. Local political spatial culture also has an influence on regional courses of the COVID-19 pandemic. The study by Richter et al. [27] shows that in Germany in regions with higher proportions of non-voters and in those where far-right parties are elected as well as where the right-wing populist Alternative for Germany (AFD) party has achieved great electoral success, COVID-19 incidences have risen more sharply, with this effect occurring both in the 1st and 2nd COVID-19 waves.

### 1.2. Perception of the COVID-19 Pandemic by the Population

The successful fight against the pandemic depends to a large extent on whether the population recognizes the dangers of the pandemic, so that appropriate governmental measures can be accepted and followed. As the duration of the pandemic has extended, the visibility of conspiracy myths presenting the pandemic as the invention of powerful actors and interest groups has increased [28].

On the basis of a representative study from Germany, Hettich et al. [29] found out that the conspiracy mentality goes hand in hand with low education, low income, young age and the male sex (similar results in [28]). People living in large cities are less likely to show belief in conspiracies about the COVID-19 pandemic compared to people from rural areas [28]. In eastern states of Germany, COVID-19 conspiracy stories are more widespread than in western states [28,30].

The study of Seddig et al. [31] performed in Germany found that people with COVID-19 conspiracy beliefs are more negative about vaccination against COVID-19. This is more likely to apply to people with right-wing political attitudes, immigrants, people with low education and low incomes, young people and people with children in kindergartens and schools.

Virchow and Häusler [32] found that protests against COVID-19 measures in the German state of North Rhine-Westphalia were characterized by a heterogeneous composition and there could be found the following groups or milieus of pandemic denial, whereby these milieus are not entirely part of the conspiracy-telling pandemic denial: Esotericists, anti-vaccinationists, concerned parents, ‘Reich Citizens’-Movement (Reichsbürger), and leftists.

According to Hettich et al. [29], people with higher levels of anxiety are more prone to conspiracy mentality, with anxiety no longer having an effect on the conspiracy belief about the COVID-19 pandemic when controlling for general conspiracy mentality.

On the extent to which the conspiracy belief against the COVID-19 pandemic is widespread in Germany, one finds different information in the current research. Often the cause of this is how the COVID-19 conspiracy mentality was measured. For example, a representative online study from Spöri and Eichhorn [28] concluded that 14% of the population in Germany consider the COVID-19 pandemic to be a conspiracy myth. Respondents were asked whether they believe the coronavirus pandemic is a hoax and that the protective measures are a hysterical overreaction. On the basis of a representative study from Germany, Hettich et al. [29] found that over 40% of the German population believe that the real background of the COVID-19 pandemic will never come into the light and that the COVID-19 crisis is being talked about in a way that few individuals can benefit from it.

According to Schließler et al. [30], in addition to the general conspiracy mentality, there is also a lack of legitimacy of the political system as a predictor of COVID-19 conspiracy. Additionally, the conviction that proven behavior should not be questioned (conventionalism) and the conviction that social rules should be enforced without pity (authoritarian aggression) significantly control the belief in COVID-19 conspiracies. According to Spöri and Eichhorn [28], people who consider social media to be credible sources of information compared to traditional media are significantly more likely to believe in COVID-19 conspiracies. This would possibly be due to the “filter bubble” or “echo chamber” property of social media: People mainly hear what they want to hear through social media, because the goal of social media would not be to objectively inform people, but for them to use social media even more often [33]. According to Spöri and Eichhorn [28], during the first COVID-19 wave, being directly affected by the pandemic did not lead to major differences in belief in COVID-19 conspiracies. The assessment of the national crisis management of the Federal Government plays an important role in this: People who rate the government’s crisis management as “good” or “very good” have an 11- to 14-fold lower proportion of COVID-19 conspiracy supporters. The role of trust in government measures in relation to the COVID-19 pandemic was determined in a study by Teufel et al. [34]. At the beginning of the pandemic, it was observed how a speech addressed to the population by German Chancellor Angela Merkel was accompanied by a short-term reduction in depression and anxiety in the population.

In a study from Ghana with the participation of university students, fear of COVID-19 was positively related to psychological distress and to maladaptive coping strategies. Adaptive coping and maladaptive coping strategies had a mediating effect on fear of COVID-19 and psychological distress [35].

The differences between COVID-19 doubters and non-doubters were investigated in an online study by Teufel et al. [36]. Interestingly, COVID-19-related anxiety was equally pronounced among doubters and non-doubters. Generalized anxiety and depression were significantly higher in doubters. According to the authors, this finding could be due to the experienced loss of control in pandemic times as well as a lower level of knowledge.

## 2. Materials and Methods

The evaluation in this paper is based on the data from the SLS-Study, whose first wave was carried out in the German Democratic Republic (GDR) in 1987. The study participants were pupils of eighth grade at 41 schools in the GDR (Leipzig and Karl-Marx-Stadt). The sample was representative of the GDR birth cohort from 1973 and included 1407 young people. 587 participants agreed to continue participating in the study after the two study waves of 1988 and 1989. Since the reunification of Germany, the longitudinal study has continued to this day. This evaluation was carried out on the basis of data from the 31st and 32nd waves, corresponding to 2019 (pre-pandemic) and 2021. The field period in 2021 lasted from 11 March to 31 July, with increased infection numbers occurring approximately in the beginning to middle of that period (third wave of COVID-19). Respondents had the opportunity to participate in the survey via post with the questionnaire or online. The number of study participants in both waves was 291. Of these, about 54% were women. The average age of respondents in 2021 was 48. Approximately 58% of the people in the sample to be evaluated were married, and almost 80% had children. The largest group consisted of employees. The median net household income for the sample was 3500 to 3999 euros per month (the median value is presented as a range because it is derived from a categorical variable). In 2021, 22% of respondents were internal migrants from East to West Germany. The extent of the missing values for socio-democratic characteristics ranges from 0 to 0.3% excluding partnership (2.7%) and household income (3.4%). Detailed information on the socio-demographic composition of the sample can be found in Table A1 in the Appendix A.

Attitudes towards COVID-19 were measured with eleven indicators from the SLS wave in 2021. The measurement scale ranged from 1 to 5 for nine items and from 1 to 7 for two items with the designations for initial categories as “does not apply at all” or “do not agree at all” and for end categories as “completely apply” or “fully agree”. Information on the descriptive statistics of these items can be found in Table A2 in the Appendix A.

Mental distress was selected as an indicator of mental health and was measured with the D-Score (for validation of the questionnaire see [37]). The D-Score is measured by agreeing with the statements “I often feel depressed and discouraged”, “Sometimes I no longer know what the meaning of my life is”, “I am often at a loss, I no longer understand the world” as well as with the question “Are you afraid of the future?”. Answer options are 1 “yes, and for years”, 2 “yes, but only for a few months” and 3 “no”. Information on the D-Score is available for measurement points 2019 and 2021, distribution of corresponding items can be found in Table A3 in the Appendix A.

To measure subjective physical health, the screening instrument called the G-Score developed by Schneider et al. [38] was selected. Its four items ask if one has had certain complaints in the last 12 months. Specifically, “nervousness”, “stomach complaints”, “insomnia” and “heart problems” are measured, for which respondents could choose whether complaints 1 “yes, frequently”, 2 “yes, from time to time”, 3 “yes, rarely” or 4 “no, never” occurred. The G-Score was also collected in both 2019 and 2021. Distribution of its items can be found in Table A4 in the Appendix A.

The socio-demographic factors were measured by the variables “gender”, “household net income”, “occupational status”. Age was not taken into account in the calculations as the sample was age homogeneous. The household net income was measured using a scale that ranged from the lowest level 0 “no income” in 500 euro intervals to the category “5000 euros and more”. Gender was queried with the categories “female”, “male” and “diverse”, whereby the category “diverse” was not covered in the survey. The occupational status was measured by the categories “worker”, “employee”, “self-employed”, “civil servant”, “housewife/househusband/parental leave”, “unemployed” and “something else”. Due to the small number of cases, the last three categories were combined in the model calculations as the category “no work/other”. In addition to the socio-demographic characteristics, the status as an internal migrant was also considered as an explanatory variable. Studies show that internal migrants report more distress and somatoform complaints than East or West Germans [39].

First, the items used to measure COVID-19 attitudes were subjected to exploratory factor analysis to understand the dimensionality of the attitudes and reduce the majority of the items to a few factors underlying the items. For this purpose, an Exploratory Structural Equation Modeling (ESEM) was carried out. The structure of the attitudes to COVID-19 determined in this way was then checked in a simultaneous confirmatory factor analysis (for the procedure see [40]).

We carried out simultaneous confirmatory factor analysis with the program Mplus (version 7.3, [41]) to test the whole underlying measurement model and possible cross-loadings and error correlations between the items of different constructs [40]. We used the χ^2^-test, the Comparative Fit Index (CFI), the Tucker–Lewis Index (TLI), the Root-Mean-Squared Error of Approximation (RMSEA), and the Standardized Root-Mean-Squared Residual (SRMR) to evaluate the model fit based on their traditionally recommended cut-off values: the *p*-value of the χ^2^-test should be >0.05 (0.01), CFI/TLI > 0.97 (0.95), RMSEA smaller than 0.05 (0.08) and SRMR smaller than 0.05 (0.10), to indicate a good (acceptable) fit between the theoretical model and empirical data [42,43].

**Figure 1:**
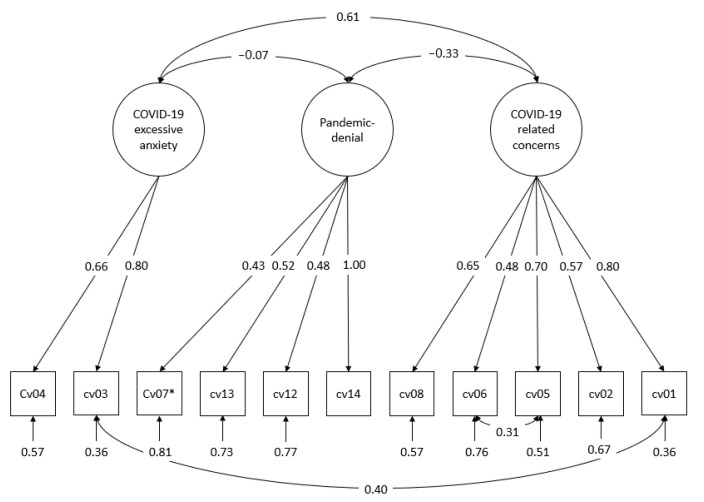
Standardized solution of the simultaneous confirmatory factor analysis for the dimensions of COVID-19 attitudes, MLR estimation. χ^2^ = 82.287 (df = 40, *p* < 0.001), RMSEA = 0.057, CFI = 0.952, TLI = 0.934, SRMR = 0.054. cv01: I am afraid of becoming infected with COVID-19; cv02: I feel like I have little control over whether or not I get infected with COVID-19; cv03: I feel like I would not survive a COVID-19 infection; cv04: I feel like I have to give up because of COVID-19; cv05: I am afraid of infecting others with COVID-19; cv06: My family and friends are afraid that they will get infected by me with COVID-19; cv07 *: The German government has done the right things in dealing with COVID-19; cv08: COVID-19 worries me; cv12: COVID-19 is not much worse than the annually recurring flu; cv13: The actual background of the COVID-19 disease will never come to light; cv14: The COVID-19 crisis has been talked about in such a way that few can benefit from it. * The item has been inverted so that high values represent a negative attitude. Observed variables are represented by rectangles and latent variables by circles. Arrows directed from the latent factors to the measurement indicators represent factor loadings. Arrows directed at measurement indicators without “emitting” variables represent the measurement error of the respective indicator. Curved two-directional arrows correspond to undirected relationships, i.e., covariances or correlations either between latent factors or between measurement errors. Standardized factor loadings greater than or equal to 0.30 or 0.40 can be interpreted as salient [40].

We used the maximum likelihood with robust standard errors (MLR) estimator for the measurement model of COVID-19 attitudes. The advantage of this method is its robustness against non-normally distributed data. The standard errors of the parameter estimators as well as the χ^2^ statistics were corrected for non-normality [40]. Due to a Heywood case [44] we fixed the residual variance of the corresponding item (cv14, see Figure 1) to 0 in the measurement model of COVID-19 attitudes.

To test the assumption that health variables have an impact on COVID-19-related attitudes, we first tested the stability and interaction between mental distress and subjective physical health in the period before the COVID-19 pandemic (2019) and during the pandemic (2021). For this purpose, it should first be checked whether there is a measurement invariance of the two factors between two measurement times (for the procedure see [45]). We used the WLSMV estimator with theta-parameterization [46] for the measurement models of the health variables since the indicators were ordinal, the sample size was small and the data were non-normally distributed [47]. We tested the longitudinal measurement invariance of both factors and then checked their stability over time as well as their mutual influences on each other in a cross-lagged model. After these tests, we added the factors used to measure COVID-19 attitudes to the cross-lagged model of health variables, while also controlling for socio-demographic characteristics with the specification of their effects on health variables.

## 3. Results

### 3.1. Dimensionality of COVID-19-Related Attitudes

The structure of COVID-19 attitudes, which we determined in an exploratory factor analysis and then checked in a simultaneous confirmatory factor analysis, is as follows (see Figure 1). Three latent factors were identified. The first factor, measured with five items, could be interpreted in the sense of concerns related to COVID-19. At higher levels of this factor, respondents are afraid of infecting themselves and others with COVID-19 and are worried about it. The second factor could be interpreted in the sense of pandemic denial, whereby conspiracy belief is also included: With higher values on this factor, one thinks that “COVID-19 is no worse than the annually recurring flu”, that it is “only talked about so that few can benefit from it”, and that “the real background of this disease will never come to light”. Dissatisfaction with the work of the German government in dealing with COVID-19 is also positively associated with this attitude. In the factor analyses, a third factor was also extracted. With higher values for this factor, one has the feeling of having to give up because of COVID-19 or not surviving the disease. We interpreted the third factor as excessive COVID-19 anxiety, which is distinguishable from “typical” concerns related to COVID-19.

Based on the modification indices we specified a residual correlation between two item pairs. The first item pair with a residual correlation is “I am afraid of infecting others with COVID-19” (cv05) and “My family and friends are afraid that they will get infected by me with COVID-19” (cv06). Apparently, the fear of infecting others correlates with the perceived fear of being infected by others. The second item pair with a residual correlation is “I feel like I would not survive a COVID-19 infection” (cv03) and “I am afraid of becoming infected with COVID-19” (cv01): The feeling of not surviving the COVID-19 disease goes hand in hand with the fear of becoming infected.

After these two modifications, we obtained a satisfactory fit solution for the model (χ^2^ = 82.287 (df = 40, *p* < 0.001), RMSEA = 0.057, CFI = 0.952, TLI = 0.934, SRMR = 0.054). Intuitively, COVID-19-related concerns and excessive COVID-19 anxiety correlated strongly with each other (r = 0.61). Moderately strong and negative was the link between pandemic denial and COVID-19-related concerns: People who were afraid to be infected with COVID-19 were less inclined to downplay the pandemic and vice versa (see Figure 1).

### 3.2. Stability and Interaction between Psychological Distress and Subjective Physical Health over Time

When studying the effects of psychological distress and subjective physical health over time, the first step is to ensure that the same constructs are measured identically at both measurement times. In other words, the longitudinal measurement invariance of the G-Score and D-Score between 2019 and 2021 has to be tested. This was carried out step by step, whereby for each new model the χ^2^-difference test relative to the previous model was also calculated. The fit statistics of the model calculations made for this purpose can be found in Table 1.

In the first step, the baseline model was calculated, in which the assumption was tested that the same pattern of factor loadings lasts over time. Factor latent means were fixed to zero at the first time and the difference from the first measurement point was measured at the second point. An autocorrelation of the residuals of the measurement indicators was specified. The fit statistics indicated an excellent solution. In the second step, the loading invariance was tested, in which factor loadings of the constructs were equated in both measurement times. The χ^2^-difference test did not become significant, which means that by equating the factor loadings, the model fit did not deteriorate. In the third step, the threshold invariance was tested, in which the assumption was tested that the threshold level of going from one response category to the next is identical over time [46]. Again, we had no significant deterioration of the model fit after equating the corresponding thresholds for all items. In the fourth step, the unique factor invariance was tested, in which all unique factor variances were held equal over time (fixed to 1.0). In this model, the χ2-difference test was significant at the 5% error level, but the change in the CFI value compared to the previous model was only −0.003, which is an indicator that this model did not deteriorate compared to the previous model [48] and thus the unique factor invariance can be considered confirmed.

After the longitudinal measurement invariance of the G-Score and D-Score was confirmed, autoregressive and cross-lagged paths were added to the model. A good fit solution could also be achieved for this model (χ^2^ = 153.198, (df = 116, *p* = 0.012), RMSEA = 0.030, CFI = 0.992, TLI = 0.992).

The following observations could be made using the cross-lagged model:

The psychological distress from 2019 leads to distress and poorer subjective physical health in 2021. Earlier poor health leads to poor health after two years but has no effect on later distress.

The differences in latent factor mean values between two measurement points were not significant for either construct (0.210 for the D-Score and −0.112 for the G-Score). Based on these observations, it can be concluded that the group values for the D-Score and G-Score remained stable from 2019 to 2021.

### 3.3. Influence of D-Score and G-Score on COVID-19 Attitudes

Due to the complexity of the further models and the small sample size, the decision was made to include the G-Score and D-Score as averages of the corresponding items in the further analysis. We used the MLR method for the estimation.

In a common model, three factors of COVID-19 attitudes were added to the cross-lagged model of the health variables. As mentioned above, the D-Score and G-Score were specified as averages of the corresponding items. Subjective physical health (G-score) and psychological distress (D-score) at the 2nd time (2021) acted as determinants of COVID-19 attitudes, but were in turn explained by health variables in the 1st measurement point (2019).

**Figure 2:**
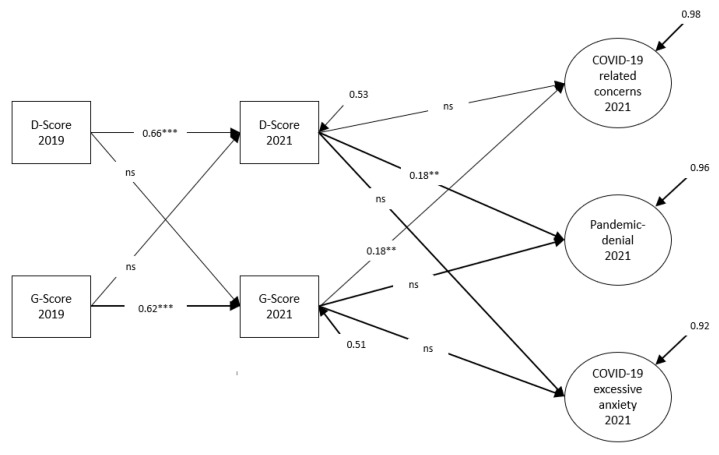
Cross-lagged model to explain COVID-19 attitudes (standardized solution), MLR estimation. χ^2^ = 117.925 (df = 78, *p* = 0.002), RMSEA = 0.038, CFI = 0.969, TLI = 0.959, SRMR = 0.046. **, *** significant at the 5% or 1% level. The measurement model of COVID-19 attitudes as well as (residual) correlations between the factors are not shown here. Observed variables are represented by rectangles and latent variables by circles. Direct effects are depicted by unidirectional arrows. Arrows without “emitting” variables represent the measurement error of the respective variable.

This model showed a good fit to the data. The findings on the influence of health variables on COVID-19 attitudes are shown in Figure 2.

The results showed that COVID-19-related concerns depended on subjective physical health. Pandemic denial, on the other hand, was determined by psychological distress. The effects of health variables on excessive COVID-19 anxiety were not significant at the 5% error level. Additionally, the influence of previous distress on later subjective physical health was not significant at the 5% error level.

### 3.4. Role of Socio-Demographic Characteristics

**Figure 3:**
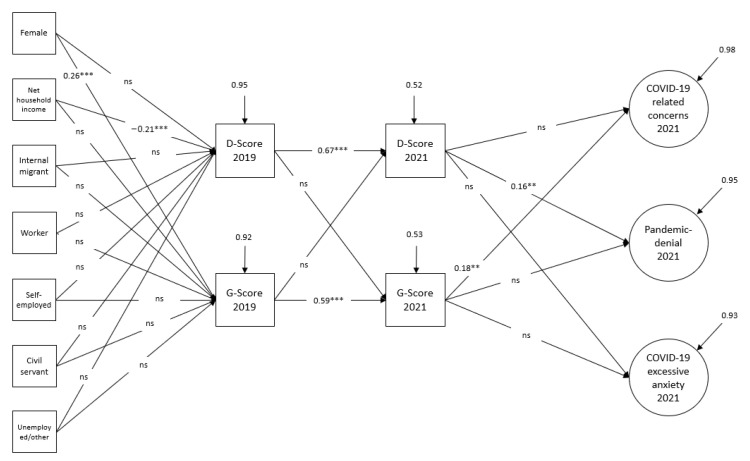
Influence of socio-demographic variables on health variables in the cross-lagged model to explain COVID-19 attitudes, MLR estimation. χ^2^ = 254.304 (df = 169, *p* < 0.001), RMSEA = 0.041, CFI = 0.936, TLI = 0.921, SRMR = 0.057. **, *** significant at the 5% or 1% level. The measurement model of COVID-19 attitudes as well as residual correlations between the factors are not shown here. Observed variables are represented by rectangles and latent variables by circles. Direct effects are depicted by unidirectional arrows. Arrows without “emitting” variables represent the measurement error of the respective variable.

In a further step, paths for the effects of socio-demographic characteristics on health variables were specified to ensure that there were influences of health variables independent of socio-demographic variables. The effects of the variables “gender”, “household net income”, “occupational status”, “status as internal migrant” on health variables were controlled. We only considered socio-demographic variables at the 1st point in time, as stability of these variables was found between two measurement points (polychoric or Pearson’s correlation coefficients greater than 0.80). The model with socio-demographic variables showed a good model fit (see Figure 3). For the correlation matrix for all variables see the Supplementary Materials.

The effects we observed in the previous models remained unchanged.

Our data thus show that the worse the subjective physical state of health is, the greater the COVID-19-related concerns are. However, pandemic denial is influenced by psychological distress. We found no confirmation in the data that subjective physical health affects pandemic denial or that psychological distress controls COVID-19-related concerns.

In addition, the following effects of socio-demography can be seen: People with higher household incomes are more likely to report minor psychological distress. Subjective physical health, on the other hand, is gender-dependent: Women are more likely to report health problems than men. Occupational status and household income do not seem to have any influence on subjective physical health. For people who had already emigrated from East Germany at the time of 2019, no peculiarities with regard to (mental) health could be observed in comparison to the persons who had stayed in East Germany.

## 4. Discussion

This paper discusses the influence of physical and mental health on COVID-19-related attitudes. Based on the data from the Saxon Longitudinal Study, the health status of the respondents before and during the pandemic was taken into account. In contrast to a cross-sectional analysis, this allowed for a better understanding of the direction of causality.

In this study, the analysis with the autoregressive cross-lagged models using health variables showed that the latent means for subjective physical health and mental distress of the respondents remained stable between 2019 and 2021. This was also the conclusion of empirical studies that compared the situation in the pandemic with the pre-pandemic period of less than two years (e.g., [18,49]). This finding shows that respondents’ health status was not fully affected by the pandemic and thus can be included as a predictor of pandemic-related attitudes.

In accordance with previous studies, our data show that in addition to the concerns of contracting the virus, there is a perception in certain population groups that the pandemic is only talked about because of the profit of a few and that COVID-19 is no different from the annually recurring flu. However, our data also provide evidence that there are groups of people with a perception of the pandemic going beyond “typical” COVID-19-related concerns who are thus experiencing personal existential threats.

The results of our study show that people’s health affects their attitudes towards the pandemic. COVID-19-related concerns are affected by subjective physical health: With poorer health, one has greater concerns of contracting COVID-19. Mental health is more important when making judgments about the pandemic: The greater the psychological distress, the more likely the pandemic is to be downplayed or denied. However, mental distress can only determine pandemic denial to a small extent. Interestingly, existential anxiety towards COVID-19 is not affected by health variables. Other aspects, such as personality factors, probably should be taken into account here.

According to the transactional stress model of Lazarus and Folkman [4], the same stimuli do not trigger stress equally in every person. On the other hand, a pandemic can undoubtedly be considered as a life-changing critical event, posing a strong threat to one’s own security and causing, among other effects, post-traumatic stress disorder as a psychosocial consequence [15,16,17]. The further question is how to handle this stress. If the application of instrumental coping strategies is challenging in unknown circumstances, such as a pandemic, then more emotion-oriented coping strategies might come into play. Given the positive relationship between the fear of COVID-19 and intolerance of uncertainty [50], those who do not believe in COVID-19 keep their control over the situation from a subjective point of view, according to Spitzer [33]. There are also empirical findings that maladaptive coping strategies positively correlate with fear of COVID-19 [35]. Our results show that strategies such as avoidance or suppression are more likely to be used by people with higher levels of distress (similar results in [16,51]), although they did already exhibit this psychological burden before the pandemic. In unknown circumstances with limited control over the situation, this strategy may be used for self-protection by downplaying the stressor and danger.

Some implications for research can be drawn from the present study. Most often, consequences of the pandemic on human health are examined. With this study we emphasized that causality is also possible the other way around. Among other factors, subjective health also plays a role in the perception of the pandemic and subsequent behavior in this crisis situation, since behavior is controlled by personal perception. The success of fighting the pandemic is therefore also dependent on the perception of the population, so that pandemic-related measures can be accepted and followed. Further research should be conducted to understand the intervening role of the mental distress in the perception of the pandemic.

The limitations of our study are due to its small sample size and non-representativeness. Due to the age homogeneity of the sample, no conclusions can be drawn about possible effects of the age on COVID-19 attitudes. Since only one age group was studied, it cannot be excluded that the identified effects were only due to the specifics of this group. It is therefore recommended to check the findings of this study using a representative data set.

## 5. Conclusions

The results of our study show that while COVID-19 primarily affects physical health, mental well-being is no less important when attitudes about the pandemic are formed. With poor subjective physical health, one tends to be afraid of contracting the virus. With greater psychological distress, on the other hand, one tends to deny or downplay dangers of the virus and pandemic. Both strategies could be seen as personal protection mechanisms. However, in a situation like a pandemic, denial is counterproductive and can endanger both one’s own health and other people’s health. The fact that pandemic deniers show rather low approval of the COVID-19 measures of the federal government and at the same time, as noted by Teufel et al. [36], feel less informed, they should be reached even more efficiently with information campaigns in order to be able to address their fears and doubts as better as possible.

## Figures and Tables

**Table 1:** Fit statistics of the models for testing longitudinal measurement invariance of the factors G-Score and D-Score.

Modell	χ^2^ (df), *p*-Value	∆χ^2^ (df), *p*-Value	CFI	TLI	RMSEA
Configural Invariance	99.306 (90) *p* = 0.236		0.998	0.997	0.017
Loading Invariance	102.508 (96) *p* = 0.304	1.636 (6) *p* = 0.950	0.999	0.998	0.014
Threshold Invariance	118.292 (106) *p* = 0.195	17.497 (10) *p* = 0.064	0.998	0.997	0.018
Unique Factor Invariance	136.804 (114) *p* = 0.072	16.725 (8) *p* = 0.033	0.995	0.995	0.024

## Data Availability

The analysis are bases on the Saxon Longitudinal Study. The data of the Saxon Longitudinal Study are archived at the Leibniz Institute for the Social Sciences (gesis) and can be obtained for research purposes at https://search.gesis.org/research_data/ZA6249, https://search.gesis.org/research_data/ZA7841 (accessed on 20 September 2022).

## Supplementary Materials

The following supporting information can be downloaded at: https://www.mdpi.com/article/10.3390/ijerph192114538/s1. Table S1: Correlation matrix for all variables in the models explaining COVID-19 attitudes.

## Appendix A

**Table A1:** Socio-demographic characteristics of the sample (measured in 2021).

***Gender (in %)***	
Male	46.4
Female	53.6
Age (mean)	48.4
**Children (in %)**	79.7
**Number of children (mean)**	1.79
***Family status (in %)***	
Married living together	55.0
Married living separately	3.4
Single	29.9
Divorced	10.0
Widowed	1.7
**Steady partner (in %)**	79.9
***Occupational status (in %)***	
Worker	19.7
Employee	60.3
Self-employed	6.9
Housewife/househusband/parental leave	1.0
Civil servant	6.9
Unemployed	2.4
Other	2.8
**Net household income in € (median) ***	3500 to 3999
***Migration background (in %)***	
Immigrated to West Germany	21.7
Immigrated abroad	2.7
Lives in East Germany	75.5

N = 291, measured in 2021. * The median value is presented as a range because it is derived from a categorical variable.

**Table A2:** Descriptive statistics of variables to measure attitudes to COVID-19 (measured in 2021).

	Minimum “Disagree”	Maximum “Agree”	Mean	Standard Deviation	Skewness	Kurtosis	N
I am afraid of becoming infected with COVID-19.	1	5	2.87	1.263	0.034	−1.236	291
I feel like I have little control over whether or not I get infected with COVID-19.	1	5	2.87	1.204	0.094	−1.019	289
I am afraid of infecting others with COVID-19.	1	5	2.52	1.280	0.347	−1.141	289
My family and friends are afraid that they will get infected by me with COVID-19.	1	5	2.09	1.063	0.709	−−0.380	289
COVID-19 worries me.	1	5	3.38	1.203	−0.572	−0.686	290
COVID-19 is not much worse than the annually recurring flu.	1	5	2.54	1.318	0.470	−0.941	290
I feel like I have to give up because of COVID-19.	1	5	1.46	0.835	1.920	3.244	291
I feel like I would not survive a COVID-19 infection.	1	5	1.87	0.939	1.025	0.682	291
The German government has taken the right steps in dealing with COVID-19.	1	5	2.51	1.212	0.238	−1.218	290
The COVID-19 crisis has been talked about in such a way that few can benefit from it.	1	7	3.90	2.123	−0.017	1.325	290
The actual background of the coronavirus disease will never come to light.	1	7	4.89	2.054	−0.652	−0.895	291

Some of the items were adapted in their wording from Wu et al. [52]. The other items are self-formulated.

**Table A3:** Distribution of items for measuring D-Score in percent, N = 291.

	1 “No”	2 “Yes, for a Few Months Now”	3 “Yes, for Years”	Missing
*Measured in 2019*				
I often feel depressed and discouraged.	77.0	11.0	11.7	0.3
Sometimes I do not know what the meaning of my life is anymore.	89.3	3.1	6.9	0.7
I am often at a loss; I no longer understand the world.	83.2	6.5	7.6	2.7
Are you afraid of the future?	74.9	13.7	10.7	0.7
*Measured in 2021*				
I often feel depressed and discouraged.	75.3	14.1	10.3	0.3
Sometimes I do not know what the meaning of my life is anymore.	88.3	6.2	5.2	0.3
I am often at a loss, I no longer understand the world.	81.1	11.3	7.2	0.3
Are you afraid of the future?	72.2	19.6	7.6	0.7

**Table A4:** Distribution of items for measuring G-Score in percent, N = 291.

	1 “Never”	2 “Seldom”	3 “From Time to Time”	4 “Frequently”	Missing
*Measured in 2019*					
Nervousness	30.2	30.6	26.8	11.3	1.0
Stomach trouble	49.8	25.1	18.6	5.5	1.0
Insomnia	25.1	29.6	30.6	14.8	0.0
Heart trouble	68.4	15.8	11.3	2.4	2.1
*Measured in 2021*					
Nervousness	35.4	27.8	26.5	8.9	1.4
Stomach trouble	54.6	20.3	17.5	7.2	0.3
Insomnia	31.6	25.4	27.5	14.4	1.0
Heart trouble	67.0	17.5	11.3	2.7	1.4
