# Supplementary material for: Are Attitudes towards COVID-19 Pandemic Related to Subjective Physical and Mental Health?
Source: Int J Environ Res Public Health. 2022 Nov 5;19(21):14538. doi: 10.3390/ijerph192114538 (PMC9656892; doi:10.3390/ijerph192114538)
Supplement: Supplementary file 1 [file ijerph-19-14538-s001.zip › ijerph-1955948-supplementary.pdf]

Table S1: Correlation matrix for all variables in the models explaining COVID-19 attitudes.

	CV01	CV02	CV05	CV06	CV08
CV01	1.000				
CV02	0.463	1.000			
CV05	0.561	0.447	1.000		
CV06	0.365	0.301	0.534	1.000	
CV08	0.523	0.357	0.409	0.343	1.000
CV14	-0.318	-0.026	-0.217	0.003	-0.321
CV12	-0.184	0.041	-0.101	-0.031	-0.120
CV13	-0.105	0.125	-0.084	0.015	-0.096
CV03	0.522	0.327	0.328	0.324	0.335
CV04	0.266	0.233	0.304	0.313	0.262
Female	0.042	0.014	0.103	0.005	0.090
Income	-0.004	-0.097	0.005	-0.042	0.151
Internal migrant	0.004	-0.095	-0.031	-0.045	-0.013
Worker	-0.090	-0.001	-0.031	0.069	-0.139
Self-employed	0.036	-0.003	0.093	0.040	0.051
Civil servant	-0.114	0.016	-0.043	-0.005	-0.016
Unemployed /other	0.027	-0.007	-0.075	-0.043	-0.073
CV07	-0.159	-0.014	-0.077	-0.007	-0.145
D_2019	0.009	0.044	-0.041	0.026	0.021
D_2021	-0.047	0.007	-0.005	0.080	0.030
G_2019	0.137	0.075	0.080	0.071	0.086
G_2021	0.091	0.078	0.077	0.110	0.082

Correlations

	CV14	CV12	CV13	CV03	CV04
CV14	1.000				
CV12	0.480	1.000			
CV13	0.515	0.214	1.000		
CV03	-0.110	-0.131	0.002	1.000	
CV04	0.021	-0.017	-0.011	0.514	1.000
Female	0.047	0.028	0.000	-0.010	0.061
Income	-0.227	-0.132	-0.063	-0.140	-0.098
Internal migrant	-0.168	-0.147	-0.143	0.037	-0.053
Worker	0.224	0.118	0.209	0.032	0.060
Self-employed	0.021	0.013	0.030	0.008	0.005
Civil servant	0.004	-0.039	-0.062	-0.069	0.103
Unemployed /other	-0.009	-0.021	-0.028	0.053	0.063
CV07	0.432	0.239	0.199	-0.053	0.001

D_2019	0.124	0.055	0.019	0.120	0.134
D_2021	0.201	0.092	0.061	0.126	0.246
G_2019	0.068	-0.010	-0.010	0.188	0.095
G_2021	0.126	0.035	0.096	0.182	0.167

Correlations

	Female	Income	Internal migrant	Worker	Self-employed
Female	1.000				
Income	-0.079	1.000			
Internal migrant	-0.070	0.117	1.000		
Worker	-0.149	-0.190	-0.068	1.000	
Self-employed	-0.044	0.127	-0.039	-0.139	1.000
Civil servant	-0.081	0.170	0.054	-0.124	-0.074
Unemployed /other	0.060	-0.249	-0.056	-0.109	-0.067
CV07	-0.028	-0.080	-0.152	0.127	0.046
D_2019	0.073	-0.190	0.014	-0.050	-0.068
D_2021	0.055	-0.248	-0.018	0.028	-0.050
G_2019	0.251	-0.099	-0.035	0.011	0.001
G_2021	0.203	-0.116	-0.029	0.011	-0.056

Correlations

	Civil servant	Unemployed/other	CV07	D_2019	D_2021
Civil servant	1.000				
Unemployed /other	-0.059	1.000			
CV07	-0.014	0.000	1.000		
D_2019	0.045	0.035	0.114	1.000	
D_2021	0.009	0.099	0.130	0.686	1.000
G_2019	-0.048	0.018	0.043	0.537	0.400
G_2021	-0.035	0.083	-0.001	0.466	0.560

Correlations

	G_2019	G_2021
G_2019	1.000	
G_2021	0.693	1.000

cv01 I am afraid of becoming infected with COVID-19.
cv02 I feel like I have little control over whether or not I get infected with COVID-19.
cv03 I feel like I would not survive a COVID-19 infection.
cv04 I feel like I have to give up because of COVID-19.
cv05 I am afraid of infecting others with COVID-19.

cv06 My family and friends are afraid that they will get infected by me with COVID-19.
cv07 The German government has done the right things in dealing with COVID-19 (inverted).
cv08 COVID-19 worries me.
cv12 COVID-19 is not much worse than the annually recurring flu.
cv13 The actual background of the coronavirus disease will never come to light.
cv14 The COVID-19 crisis has been talked about in such a way that few can benefit from it.
